# Is Maximum Food Intake in Endotherms Constrained by Net or Factorial Aerobic Scope? Lessons from the Leaf-Eared Mouse

**DOI:** 10.3389/fphys.2016.00649

**Published:** 2016-12-27

**Authors:** Karin Maldonado, Pablo Sabat, Gabriela Piriz, José M. Bogdanovich, Roberto F. Nespolo, Francisco Bozinovic

**Affiliations:** ^1^Departamento de Ciencias Ecológicas, Facultad de Ciencias, Universidad de ChileSantiago, Chile; ^2^Center of Applied Ecology and Sustainability, Pontificia Universidad Católica de ChileSantiago, Chile; ^3^Departamento de Ecología, Facultad de Ciencias Biológicas, Pontificia Universidad Católica de ChileSantiago, Chile; ^4^Instituto de Ciencias Ambientales y Evolutivas, Facultad de Ciencias, Universidad Austral de Chile, Campus Isla TejaValdivia, Chile

**Keywords:** absolute aerobic scope, assimilation capacity model, basal metabolic rate, energy metabolism, food consumption, maximum metabolic rate, muridae, rodents

## Abstract

Food availability varies substantially throughout animals' lifespans, thus the ability to profit from high food levels may directly influence animal fitness. Studies exploring the link between basal metabolic rate (BMR), growth, reproduction, and other fitness traits have shown varying relationships in terms of both magnitude and direction. The diversity of results has led to the hypothesis that these relationships are modulated by environmental conditions (e.g., food availability), suggesting that the fitness consequences of a given BMR may be context-dependent. In turn, there is indirect evidence that individuals with an increased capacity for aerobic work also have a high capacity for acquiring energy from food. Surprisingly, very few studies have explored the correlation between maximum rates of energy acquisition and BMR in endotherms, and to the best of our knowledge, none have attempted to elucidate relationships between the former and aerobic capacity [e.g., maximum metabolic rate (MMR), aerobic scope (Factorial aerobic scope, FAS; Net aerobic scope, NAS)]. In this study, we measured BMR, MMR, maximum food intake (recorded under low ambient temperature and *ad libitum* food conditions; MFI), and estimated aerobic scope in the leaf-eared mouse (*Phyllotis darwini*). We, then, examined correlations among these variables to determine whether metabolic rates and aerobic scope are functionally correlated, and whether an increased aerobic capacity is related to a higher MFI. We found that aerobic capacity measured as NAS is positively correlated with MFI in endotherms, but with neither FAS nor BMR. Therefore, it appears plausible that the capacity for assimilating energy under conditions of abundant resources is determined adaptively by NAS, as animals with higher NAS would be promoted by selection. In theory, FAS is an invariant measurement of the extreme capacity for energy turnover in relation to resting expenditure, whereas NAS represents the maximum capacity for simultaneous aerobic processes above maintenance levels. Accordingly, in our study, FAS and NAS represent different biological variables; FAS, in contrast to NAS, may not constrain food intake. The explanations for these differences are discussed in biological and mathematical terms; further, we encourage the use of NAS rather than FAS when analyzing the aerobic capacity of animals.

## Introduction

Identifying and understanding the factors driving inter-specific variation in metabolic rates is an important area of research in energetics, but also, there is increasing interest in how inter-individual variation in energy metabolism is maintained within populations (Careau et al., 2008; Burton et al., 2011; Maldonado et al., 2011). The most common estimate of the metabolic floor in endothermic animals is basal metabolic rate (BMR). Variation in BMR has been associated primarily with body mass (Kleiber, 1961), but in mammals, mass-independent BMR is also correlated with factors such as food habits, life-history strategies, productivity and temperature (see McNab, 2002 and references therein). Maximum metabolic rate (MMR), by contrast, describes the maximum aerobic output available to perform a given task, and increases in MMR have been related to high levels of thermogenesis of animals living in cold climates (Bozinovic et al., 2011; Swanson and Bozinovic, 2011). In turn, aerobic scope is a measure of aerobic capacity that describes the extent to which metabolic rate can be increased above baseline energy requirements to drive a range of key functions, including digestion, locomotion, growth, and reproduction (Guderley and Pörtner, 2010). There are two measures used for aerobic scope: factorial aerobic scope (MMR/BMR; FAS), and net aerobic scope (MMR-BMR; NAS), which have been suggested to have different biological meanings (Killen et al., 2016; Nespolo et al., 2016). Variation in aerobic scope among populations has been linked to differences in geographic distributions (Naya and Bozinovic, 2012), ability to cope with environmental extremes (Pörtner and Knust, 2007; Kassahn et al., 2009), and migratory effort (Tudorache et al., 2007; Eliason et al., 2011), suggesting that it may be both a trait of ecological relevance and a measure of aerobic capacity.

The existence of intra-specific variation in metabolic rates among individuals within populations is well established (Careau et al., 2008, 2009; Maldonado et al., 2012). Consistent individual differences in metabolic rates and activity levels have been in general, related to food intake rates, growth and/or fecundity and other life-history traits in a wide range of taxa (Biro and Stamps, 2008). In particular, studies attempting to elucidate the relationship between BMR and fitness have shown varying responses, in terms of the both its magnitude and direction (Careau et al., 2014). The diversity of results has led to the idea that these relationships are strongly modulated by environmental conditions (e.g., food availability), highlighting the fact that fitness consequences of physiological variation are context-dependent (Burton et al., 2011; Careau et al., 2014). These arguments are closely related to the results of Auer et al. (2015a), who found that the association between maintenance costs and aerobic scope in ectotherms, is strongly modulated by food availability. Furthermore, Auer et al. (2015b) propose that individuals with comparatively higher aerobic scope may be favored by selection due to their ability to capitalize reserves during periods of high food availability.

Several lines of research suggest that individuals with high capacity for aerobic work, also have high capacity for acquiring energy from food. For instance, some authors have explored digestive and metabolic flexibility under regimes that represent an increase in energy demands (see peripheral versus central limitations hypothesis; Hammond and Diamond, 1997). Others refer to the relationship between metabolism and energy assimilation, evidencing a range of energetic strategies within populations, in which, for example, individuals with high/low energy expenditure and high/low assimilation capacities exhibit different selective regimes (Careau et al., 2008; Gaitan-Espitia and Nespolo, 2014; Bartheld et al., 2015). Finally, different mice strains selected from low and high-energy expenditure have shown correlated responses to selection in metabolically active organ masses, especially with intestine mass (Konarzewski and Diamond, 1995; Konarzewski et al., 2000; Selman et al., 2001). Nevertheless, studies that measure maximum rates of energy intake and aerobic capacity directly are particularly rare (Song and Wang, 2002; Auer et al., 2015b).

Given that food availability varies substantially throughout the course of animal's lifespans, the ability to profit from high food levels may directly influence growth, reproduction and other fitness related traits. Assessing the generality of this capacity in endotherms is important, since their energy requirements are about 30 times larger than those of a comparable sized ectotherm (Careau et al., 2014). Accordingly, we used the leaf-eared mouse to test: (i) if metabolic rates and aerobic capacity (NAS and FAS) are functionally correlated, and (ii) whether an increase in aerobic capacity (NAS and FAS) is related to a higher capacity for food acquisition (measured as maximum food intake, MFI).

## Materials and methods

### Animals and study site

**Figure 1:**
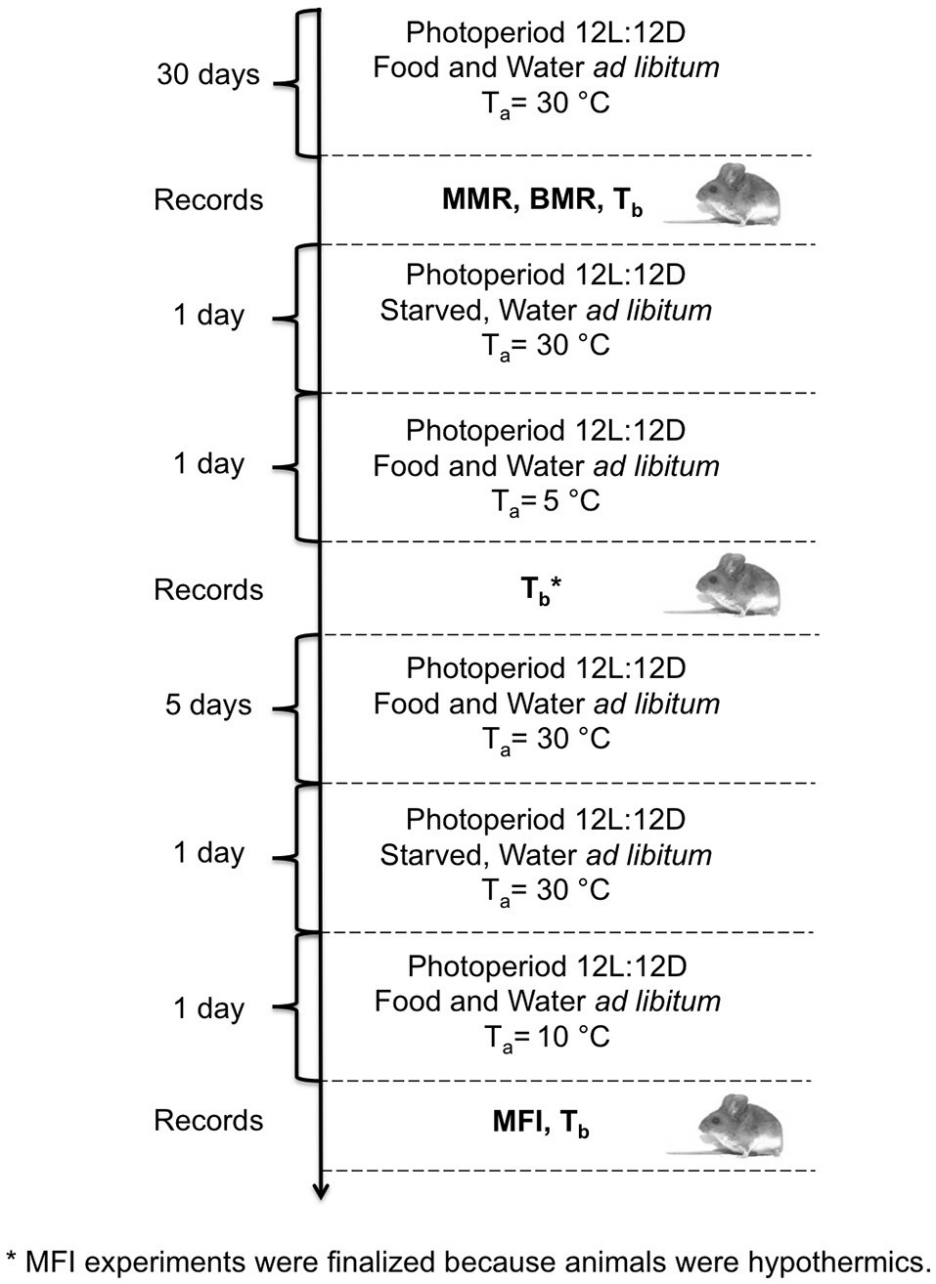
**Schematic diagram and timeline of experimental procedures performed in *P. darwini***. BMR measurements were performed at 30° and MMR at < 5°C. Maximum food intake was measured at 10°C. After all measurements T_b_ was recorded.

We used the leaf-eared mouse *Phyllotis darwini* (Muridae) as a model. *P. darwini* is a nocturnal rodent that feeds primarily on seeds, but also consumes insects and grasses (Bozinovic et al., 2007). Twenty-two non-reproductive adult rodents (weight: 49.88 ± 13.5 g) were captured using Sherman traps in a southern temperate region of Chile (36° 12′S, 72° 39′W) during the austral winter (June-July) of 2015. After capture, animals were weighed with an electronic balance (± 0.1 g), and transported to the laboratory in Santiago, Chile (33° 27′S, 70° 42′W). For identification and to measure body temperature (T_b_) during the experiments, subcutaneous transponders (IPTT-300, BioMedic Data Systems, Seaford, DE), with an accuracy of ± 0.2°C in the calibrated range of 32–43°C, as well as a handheld reader (DAS-6006/7 Smart Probe, Bio Medic Data Systems Inc.) were used. Transponders were implanted subcutaneously in the lower back using a sterilized needle assembly provided by the manufacturer (BioMedic, Seaford, DE), for which anesthesia was not required. After this procedure, rodents were allowed to recover and then returned to their individual plastic cages of 35 × 25 × 15 cm. All animals were maintained for 6 weeks at 30 ± 2°C with a photoperiod of 12L:12D and fed with commercial rabbit food pellets (Champion S. A., Santiago, Chile) and water *ad libitum* prior to BMR, MMR and MFI measurements (Figure 1).

### Basal metabolic rate

Basal metabolic rate was determined as the minimum oxygen consumption measured in non-reproductive adult animals, during their inactive phase (8:00 h–17:00 h), under post-absorptive conditions in a thermoneutral environment, using a standard flow-through respirometry system (Nespolo et al., 2003a). First, rodents were weighed using an electronic balance (± 0.1 g), placed in a acrylic metabolic chamber (1 L) and then placed in a controlled temperature cabinet (Sable Systems, Henderson, NV, USA) at 30 ± 0.5°C, which is within the thermoneutral zone of this species (Bozinovic and Rosenmann, 1988; Bozinovic et al., 2007). The metabolic chamber received air that passed through CO_2_/water absorbent granules (Baralyme and Drierite, respectively) at 750 mL min^−1^ from a mass flow controller, which was enough to ensure adequate mixing in the chamber. The excurrent air passed again through columns of Baralyme, and Drierite before passing through an O_2_-analyzer, model Foxbox (Sable System, Nevada, USA), which was calibrated with a known mix of oxygen (20%) and nitrogen (80%) certified by chromatography (INDURA, Chile). Air was sampled every 5 s by the O_2_ analyzer. The mass flow meter of the Foxbox was calibrated monthly with a volumetric flow meter. Rodents remained in the metabolic chamber for at least 8 h until visual inspection of recorded data determined that steady-state conditions were reached. Colonic and transponders T_b_ were recorded at the end of each measurement using a Digi-Sense copper-constant thermocouple. Since water vapor and CO_2_ were scrubbed before entering the O_2_ analyzer, the oxygen consumed per hour, was calculated according to Withers (1977): VO_2_ = [FR^*^60^*^(Fi O_2_ − Fe O_2_)]/(1 − Fi O_2_), where FR is the flow rate in mLmin^−1^, and Fi and Fe are the fractional concentrations of O_2_ entering and leaving the metabolic chamber, respectively. Output from the oxygen analyzer (%) and the flow meter were digitalized using a Universal Interface II (Sable Systems, Nevada, USA) and recorded on a personal computer using EXPEDATA data acquisition software (Sable Systems, Nevada, USA). To estimate BMR, we averaged O_2_ concentration of the excurrent air stream over a 10 min period after steady state was reached (Tieleman et al., 2002).

### Maximum metabolic rate

Maximum metabolic rate was estimated as the maximum oxygen consumption elicited in response to cold exposure in a He-O_2_ (helox) atmosphere according to the procedure of Rosenmann and Morrison (1974), using a standard flow-through respirometry system as described by Chappell and Bachman (1995) and Swanson and Bozinovic (2011). The mixture of He (80%) and O_2_ (20%) was certified by chromatography (INDURA, Chile). In brief, animals were weighed using an electronic balance (± 0.1 g), placed in a acrylic metabolic chamber fitted with 2 cm thick rubber foam on the floor to prevent direct contact between the animal and the cold acrylic surface, and then placed within a controlled temperature cabinet. The chamber temperature was monitored continuously with a Digi-Sense copper-constant thermocouple inserted into the outlet port of the metabolic chamber. All MMR trials started with a temperature of 5°C, while the temperature cabinet was reduced (3°C /20 min). The mixture of He (80%) and O_2_ (20%) was supplied by a volumetric flow meter (before entering the metabolic chamber) at 1002 ± 3 mLmin^−1^ (Nespolo et al., 2003b). As with BMR measurements, air passed through granules of Baralyme and Drierite after passing through the chamber. Also, T_b_ from the transponder was scanned continuously using a handheld reader coupled to the metabolic chamber. VO_2_ was measured at 5 s sampling intervals. The end-point of MMR experiments was hypothermia, a reliable indicator that subjects have reached maximal heat production levels (Nespolo et al., 2003b). Hypothermia was confirmed by records of T_b_ in real time (~ 10°C below relative to its value at thermoneutrality), which is accompanied with decrement changes in T_a_ with no further increase in VO_2_. The duration of MMR measurements depended on animals' body mass, but was in all cases less than 1 h and typically less than 30 min. Animals were subsequently removed from the chamber, and colonic T_b_ was measured immediately in order to corroborate hypothermia. Then, animals were warmed and observed until reaching normothermia. Similar to BMR measurements, output from the oxygen analyzer (%) and the flow meter were digitalized using a Universal Interface II (Sable Systems, Nevada, USA) and recorded on a personal computer using EXPEDATA data acquisition software (Sable Systems, Nevada, USA). We used the highest 5 min of VO_2_ consumption as a measure of MMR.

### Maximum food intake

After oxygen consumption measurements (Figure 1), maximum food intake was estimated for each individual, as the food intake at low ambient temperature (10 ± 2°C) and under *ad libitum* food (Koteja, 1995). Prior to MFI experiments, all animals were fasted for 24 h. At the beginning of MFI experiment, animals were weighed with an electronic balance (± 0.1 g) and then housed individually (without bedding) in a plastic cage (25 × 25 × 25 cm) at an ambient temperature of 10 ± 2°C, and a photoperiod of 12L:12D. A weighed amount of food and water *ad libitum* was then provided in metal feeders for 24 h (rabbit pellets, Champion S.A., Chile, energy content = 17 kJ g^−1^). T_b_ was scanned using a handheld transponder reader 4 and 8 h after the initiation of the experiments. We selected an ambient temperature of 10 ± 2°C to perform MFI experiments, because in a previous trial at 5 ± 2°C animals become hypothermic (T_b_ = 33.1 ± 4.5°C, Figure 1). By contrast, when we measured MFI at 10 ± 2°C all animals were normothermic (T_b_ = 36.1 ± 0.9°C).

To measure food intake, once the MFI experiment ended, animals were removed from their cages, and the amount of remaining food, including the food that was spilled on the floor of the cage, was collected and weighted with an electronic balance (± 0.01 g). Then, MFI was calculated as the mass (in grams, adjusted for moisture) of food provided less that recovered and converted to energy units (joules) multiplying by the energy content of pellets.

We calculated FAS and NAS as MMR/BMR and MMR-BMR, respectively. We used 20.08 J ml^−1^ O_2_ to convert oxygen consumption to heat production (Schmidt-Nielsen, 1997). All experimental procedures were carried out in accordance with the recommendations of the guide “Regulation of the use and care of experimental animals” of the Bioethics Committee, Comisión Nacional de Investigación Científica y Tecnológica (CONICYT). The protocol was approved by the Institutional Animal Care Committee of the University of Chile. Animals were captured with permit from The National Wildlife Service (Servicio Agrícola Ganadero, N° 3895/2015).

### Statistics

We tested the relationships between body mass, BMR, MMR, NAS, FAS, and MFI using a Generalized Additive Model (“gam” package in R). We used this test due to the non-linear relationship between the measured variables. Also, we assumed a Gaussian distribution with an identity function as a link. To remove the effect of body mass, we calculated the residuals (r) of BMR, MMR, NAS, FAS, and MFI for analysis. Residuals were calculated from the regression equations between each variable and body mass. Then, residuals from the relations (rBMR, rMMR, rNAS, rFAS, and rMFI) correspond with estimators of the mass-independent variables, which were used in the subsequent analysis. The relationship between residuals were analyzed using Mixed GAM Computation Vehicle with GCV/AIC/REML Smoothness Estimation (mgcv). In a generalized additive model, when using a smoothing splines to estimate a function, the user faces the problem of choosing the smoothing parameters. The use of an effective degree of freedom (Edf) corresponding to a penalized degree of freedom allows coping this challenge (Wood, 2006). Thus, models were fitted using penalized spline, and evaluated with the Generalized Cross Validation (GCV) method (Hastie and Tibshirani, 1990). The output of the generalized additive models included the percentage of explained deviance by models (Dev. Expl.) and the effective degree of freedom (Edf) for each term analyzed. We set the significance level at α = 0.05. Unless otherwise stated, values are presented as mean ± SD. All statistical analyses were performed using the “R” platform, version 2.12.2 (R Development Core Team, 2012).

## Results

In relation with metabolic rates, the values for BMR and MMR were in average 1.38 ± 0.3 and 5.63 ± 1.4 kJ h^−1^, respectively. At the end of BMR and MMR measurements, T_b_ was 36.9 ± 0.8° and 26.4 ± 1.9°C, respectively. With respect to aerobic scope, FAS was in average 4.12 ± 0.88, whereas NAS was 4.25 ± 1.3 kJ h^−1^. Rodents food intake during MFI experiment was in average 0.463 ± 0.12 g h^−1^, which was equivalent to 7.87 ± 2.04 kJ h^−1^.

**Figure 2:**
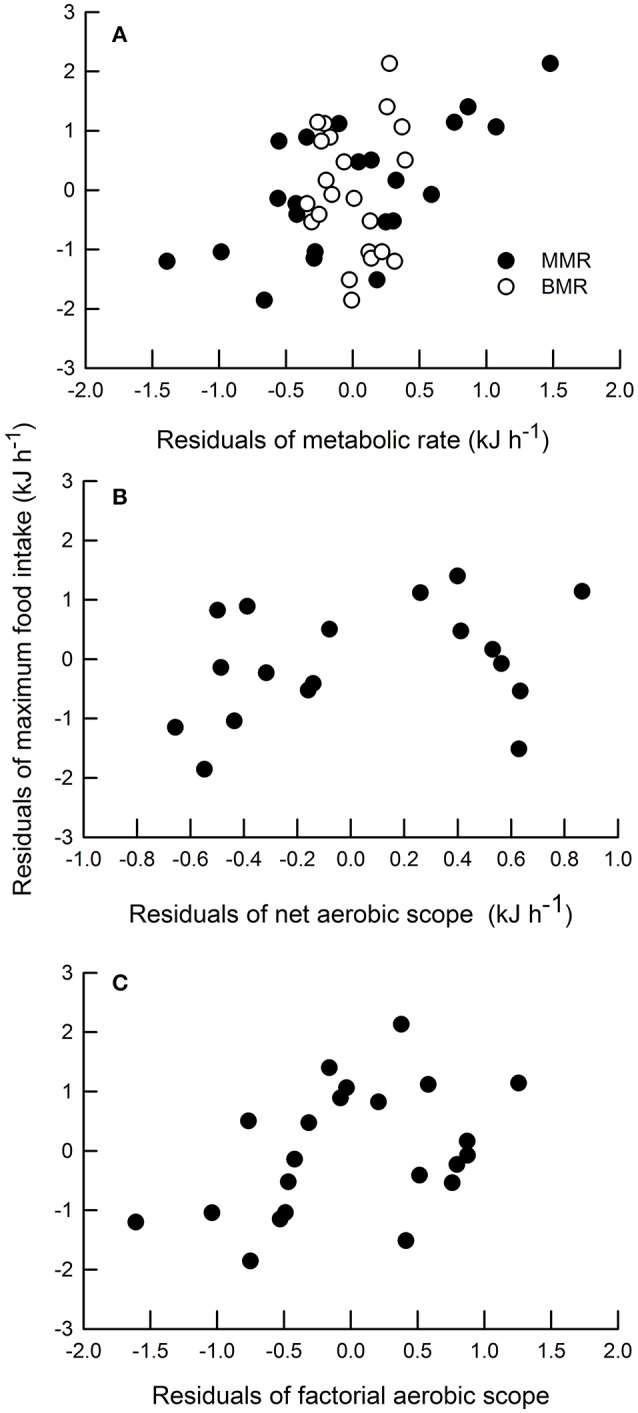
**Correlation between residuals of maximum food intake and (A)** residuals of metabolic rate (MMR = black circles, *R*^2^ = 0.39, *P* = 0.01; BMR = white circles, *R*^2^ = 0.19, *P* = 0.2); **(B)** residuals of net aerobic scope (*R*^2^ = 0.31, *P* = 0.004); and **(C)** residuals of factorial aerobic scope (*R*^2^ = 0.28, *P* = 0.124) in *Phyllotis darwini*.

As expected, most of physiological variables in a whole-animal basis, exhibited significant associations with body mass. In fact, positive correlations were detected between body mass and BMR, MMR and NAS (Table 1A). Moreover, MFI also showed a significant positive relationship with body mass (Table 1A). However, the relationship between body mass and FAS was non-significant (Table 1A). A non-significant relationship between the residuals (against body mass) of BMR and MMR was observed (Table 1B); similarly, the relationship between the residuals of BMR and the residuals of NAS was non-significant (Table 1B). In turn, a significant positive relationship between the residuals of BMR and the residuals of FAS was detected (Table 1B). Regarding maximum rates of energy expenditure, we found a positive correlation between the residuals of MMR with both measures of aerobic scope (i.e., the residuals of NAS and FAS). With respect to the relationships between metabolic variables and MFI, we found a significant correlation between the residuals of MMR and the residuals of NAS (Table 1B; Figures 2A,B). Finally the residuals of BMR and the residuals of FAS were not significantly related to the residuals of MFI (Table 1C, Figures 2A,C).

**Table 1:** **Output of the generalized additive models of: (A) the relationships among metabolic variables and maximum food intake as a function of body mass; (B) the relationships among residuals of metabolic variables and (C) relationships between residuals of metabolic variables and food intake**.

	**Edf**	***F***	***P***	***R^2^* adj**	**Dev. Expl (%)**
**(A)**					
MMR	4.744	9.957	<0.001	0.72	78.9
MFI	7.44	3.774	<0.010	0.58	72.9
NAS	2.016	13.92	<0.001	0.62	65.5
BMR	1.025	12.08	<0.001	0.36	39.2
FAS	1.68	2.389	0.11	0.17	23.4
**(B)**					
rMMR vs. rNAS	1.898	56.91	<0.05	0.87	87.8
rBMR vs. rFAS	1.763	10.63	<0.05	0.52	56.0
rMMR vs. rFAS	1.891	5.11	<0.05	0.35	40.9
rBMR vs. rMMR	1.0	0.409	0.530	0.03	2.0
rBMR vs. rNAS	1.125	0.127	0.710	0.03	2.4
**(C)**					
rMFI vs. rFAS	4.002	2.143	0.124	0.28	41.7
rMFI vs. rMMR	1	14.46	0.001	0.39	42
rMFI vs. rNAS	1	10.38	0.004	0.31	34.2
rMFI vs. rBMR	3.302	1.629	0.200	0.19	32.4

*The percentage of explained deviance by models (Dev. Expl.) and the effective degree of freedom (Edf) for each term is showed. BMR, basal metabolic rate; MMR, maximum metabolic rate; NAS (net aerobic scope), MMR — BMR; FAS (factorial aerobic scope), MMR/BMR; MFI, maximum food intake; rBMR, residuals of basal metabolic rate; rMMR, residuals of maximum metabolic rate; rNAS, residuals of net aerobic scope; rFAS, residuals of factorial aerobic scope; and rMFI, residuals of maximum food intake*.

## Discussion

In this study, we essentially demonstrate that aerobic capacity is positively correlated with maximum food intake in an endotherm. According to our hypothesis, individuals with high aerobic capacity also have high capacity for energy acquisition. Interestingly, we found that MFI was correlated with NAS (and also with MMR), but not with FAS (or BMR), which may be explained by the fact that FAS is correlated with both BMR and MMR, whereas NAS demonstrated a correlation only, although strongly, with MMR. These results suggest that the extent to which metabolic rate can be increased above baseline energy requirements to drive all oxygen-consuming functions is determined primarily by the maximum power output, irrespective of maintenance requirements. Moreover, as mentioned, the high capacity for energy acquisition in cold-exposed individuals -which may determine the upper limits to energy budgets (Koteja et al., 2000)- was related to animals' capacity to increase their potential to expend energy. This prediction is pointed out in the central limitation hypothesis, in which individuals with a digestive system able to extract more energy from food would have a greater potential for sustainable metabolic rates (Weiner, 1989; Peterson et al., 1990; Hammond and Diamond, 1997; Koteja et al., 2000). Therefore, it is expectable that individuals with higher aerobic capacity also can digest food faster, and thus can consume higher amount of food (Millidine et al., 2009). Our findings are similar to those recently found in the juvenile brown trout, in which individuals with a higher NAS exhibited a higher daily maximum food intake by comparison to those with lower NAS (Auer et al., 2015b).

The aerobic assimilation model for the evolution of endothermy posits that higher long-term locomotor activity must be fuelled by an increase in food consumption, which is facilitated by an increase in the capacity of alimentary tract and visceral organs (Nespolo and Roff, 2014). Consequently, an increase in resting rates of metabolism as a by-product of aerobic capacity is anticipated (Hayes and Garland, 1995; Nespolo and Roff, 2014). In this regard, several studies have attempted to elucidate the genetic correlation between food consumption, BMR and MMR, with mixed results (Selman et al., 2001; Książek et al., 2004; Gębczyński and Konarzewski, 2009). In our study, BMR was not correlated with either MMR or MFI. This lack of functional association may be explained if MFI is associated directly with intraspecific variation in aerobic capacity or activity levels (e.g., aggressiveness, bold personality types; for a review see Biro and Stamps, 2008) rather than with traits related to maintenance requirements. Experimental studies are needed to evaluate whether differences in rates of energy expenditure and food intake are related, for example, to constitutive differences in hormonal or metabolic intensity of animal tissues (see Breslow et al., 1999; Speakman, 2013). The current paucity of research exploring these comparisons limits generalization of our findings, highlighting the need for improved understanding of the relationship between aerobic capacity and traits related to activity levels.

On the other hand, measurement of FAS and NAS as a metric of aerobic capacity has received varied treatments. Authors working with ectotherms have traditionally used and interpreted NAS, whereas those working with endotherms use FAS (Nespolo et al., 2016). In addition, it has been suggested that FAS and NAS are variables that lead to different biological conclusions (Killen et al., 2016; Nespolo et al., 2016). For instance, an interspecific study involving 148 endotherm and ectotherm species revealed that NAS is a preferable descriptor of aerobic capacity than FAS; while the former generated two evolutionary optimums that grouped endotherms separately from ectotherms, FAS was incapable of distinguishing the widely recognized differences in aerobic capacity separating the two groups (Bennett and Ruben, 1979). In our study, FAS and NAS also represent different biological variables; FAS may not constrain maximum food intake, since rFAS, contrary to rNAS, exhibited a non-significant relationship with rMFI (Figure 2C). In theory, FAS is an invariant measurement of the extreme capacity for energy turnover in relation to resting expenditure (Hinds et al., 1993; Clark et al., 2013), whereas NAS represents the maximum capacity for simultaneous aerobic processes above maintenance levels (Clark et al., 2013; Killen et al., 2014, 2016). These differences could be evidenced by the constant FAS but variable NAS (i.e., correlated with MFI) observed in our results.

An alternative mathematical explanation for the different results obtained for FAS and NAS could be related to the statistical artifact of dividing variables by different scaling exponents, the so-called “fallacy of averages” (Welsh et al., 1988). This problem refers to the fact that, when two allometric equations are divided (see differences in allometric exponent for MMR and BMR in Weibel et al., 2004; Dlugosz et al., 2013), the resulting ratio has unknown statistical properties. These problems arise from the non-linearity of each allometric equation and also from error propagation when calculating ratios (see a detailed explanation with examples in Welsh et al., 1988; Medel et al., 1995). Considering these problems and the reported results, we encourage researchers to rely on NAS rather than FAS when analyzing the aerobic capacity of animals.

Overall, our results suggest that, at an intraspecific level, MFI may be constrained by NAS in endotherms, thus reversing the classic adaptive argument assuming a limited environment (Sibley and Calow, 1986; Karasov and Martínez del Río, 2007). This has previously been suggested by Auer et al. (2015a,b) for ectotherms, and earlier by Mueller and Diamond (2001) and Bozinovic et al. (2009) for endotherms. In other words, it appears plausible that the capacity for assimilating energy under conditions of abundant resources is determined adaptively by NAS, as animals with higher NAS would be promoted by selection. Hence, endotherms with a higher NAS are better able to take advantage of changes in food availability. It is worth to note that metabolic variables usually exhibit a power function of body mass (Kleiber, 1961), therefore the strength and magnitude of the relationship between MFI and NAS could be influenced by the specific allometric exponent of a particular species (see review Tartaruga et al., 2013). Finally, metabolic measurements related to energy-demanding activities other than maintenance, on which natural selection may be operating, need to be explored in order to fully understand the link between different metabolic phenotypes and fitness.

## Author contributions

Conception and design: FB, KM. Data acquisition and analysis: JB, GP, KM. Data interpretation: FB, KM, PS, RN. All authors collaborated with the draft of the work and approved the final version of the manuscript.

### Conflict of interest statement

The authors declare that the research was conducted in the absence of any commercial or financial relationships that could be construed as a potential conflict of interest.
